# Piperacillin/tazobactam treatment in children: evidence of subtherapeutic concentrations

**DOI:** 10.3389/fphar.2024.1254005

**Published:** 2024-07-04

**Authors:** Panpan Ye, Jinyi Shi, Zixuan Guo, Xinmei Yang, Qian Li, Keguang Chen, Furong Zhao, Haiyan Zhou, Yehui Zhang, John van den Anker, Linlin Song, Wei Zhao

**Affiliations:** ^1^ Department of Clinical Pharmacy, The First Affiliated Hospital of Shandong First Medical University and Shandong Provincial Qianfoshan Hospital, Shandong Engineering and Technology Research Center for Pediatric Drug Development, Shandong Medicine and Health Key Laboratory of Clinical Pharmacy, Jinan, China; ^2^ Department of Pharmacy, China-Japan Friendship Hospital, Beijing, China; ^3^ Division of Clinical Pharmacology, Children’s National Hospital, Washington, DC, United States; ^4^ Departments of Pediatrics, Pharmacology and Physiology, Genomics and Precision Medicine, the George Washington University School of Medicine and Health Sciences, Washington, DC, United States; ^5^ Department of Paediatric Pharmacology and Pharmacometrics, University Children’s Hospital Basel, University of Basel, Basel, Switzerland; ^6^ Department of Clinical Pharmacy, Key Laboratory of Chemical Biology (Ministry of Education), School of Pharmaceutical Sciences, Cheeloo College of Medicine, Shandong University, Jinan, China

**Keywords:** piperacillin/tazobactam, concentration, pediatric, antibacterial effects, target attainment

## Abstract

**Objective:**

Piperacillin/tazobactam (PIP/TAZ) is used for the treatment of lower respiratory tract bacterial infections in children. This study was performed to evaluate if the current dosing regimen results in therapeutic drug concentrations.

**Patients and methods:**

Patients suspected or proven to have lower respiratory tract bacterial infection and administrated PIP/TAZ intravenously for a duration of no less than 0.5 h, q6h–q12h daily, were enrolled. Blood samples were collected, and PIP concentrations were determined by high-performance liquid chromatography. The individual predicted concentration of PIP was evaluated using the individual empirical Bayesian estimate method. The evaluated PK/PD targets included (1) 70% time when the predicted free drug concentration exceeds the minimum inhibitory concentration (*f*T > MIC) and (2) 50% *f*T > 4× MIC. Probability of target attainment (PTA) was assessed by the proportion of patients who reached the PK/PD targets. The PIP concentrations between different groups of patients were compared.

**Results:**

A total of 57 samples were collected from 57 patients with a median age of 2.26 years (0.17–12.58). For the PK/PD targets of 70% *f*T > MIC and 50% *f*T > 4× MIC for *Pseudomonas aeruginosa* and *Klebsiella pneumoniae*, the PTA was all 0. The median C_min_ of PIP was significantly higher in infants than in children, and the median C_min_ after administration in q8h was significantly higher than that after administration in q12h.

**Conclusion:**

The current dose regimen of PIP/TAZ leads to extremely low plasma concentrations in most children with lower respiratory tract bacterial infections. More optimized dosing regimens or better alternative therapies need to be further explored.

## 1 Introduction

Piperacillin/tazobactam (PIP/TAZ) is a frequently used beta-lactam/beta-lactamase inhibitor combination (BLBLI) with excellent tolerability (Gonçalves-Pereira and Póvoa, 2011; Jung et al., 2017). PIP/TAZ has a bactericidal effect on Gram-negative microorganisms and some Gram-positive ones (Schuetz et al., 2018; Shi and Xie, 2023), as an effective antibacterial agent to treat lower respiratory tract bacterial infection (Abramavicius et al., 2021; Mauritz, et al., 2024). Although PIP/TAZ is not recommended as a first-line treatment drug, it is still widely used in treating lower respiratory tract bacterial infections in children (Okubo, et al., 2020; Chongcharoenyanon, et al., 2021; Nyamagoud, et al., 2023).

PIP/TAZ is a time-dependent antibiotic. Its bactericidal effect is related to the time when the free drug concentration exceeds the minimum inhibitory concentration (MIC) of the pathogen (*f*T > MIC) (Delvallée et al., 2013; Zander et al., 2016). Therefore, *f*T > MIC is the best predictor of the effectiveness of PIP/TAZ. The near-maximal bactericidal effect is achieved when *f*T > MIC is be 60%–70% of the dosing interval (Drusano, 2004). Bacterial killing seems to reach its maximum when the free PIP concentration is 4–5× MIC within 50% of the dosing interval (Taccone et al., 2010; Felton et al., 2013). Therefore, 70% *f*T > MIC and 50% *f*T > 4× MIC were generally chosen as antimicrobial goals.

**FIGURE 1:**
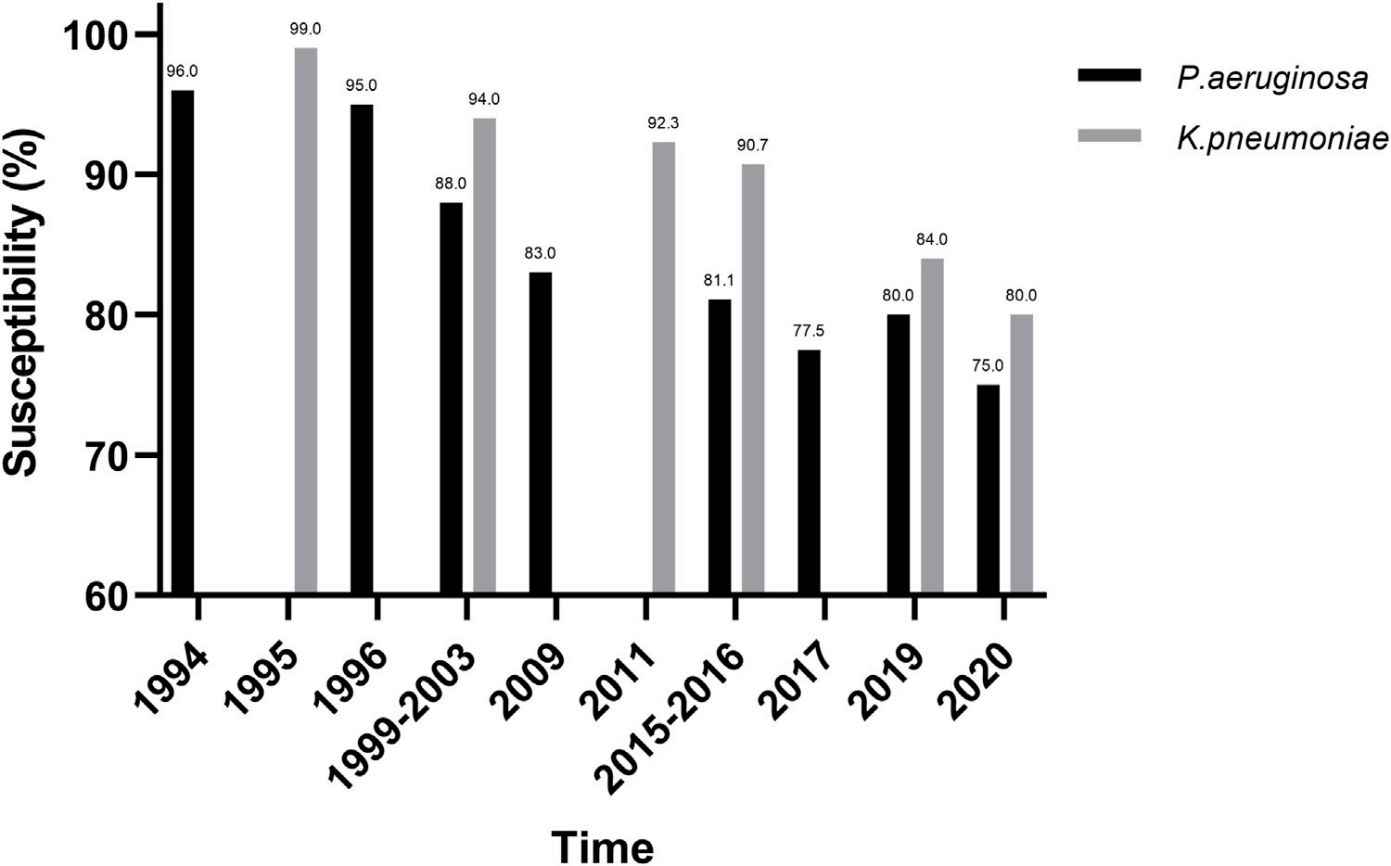
Sensitivity of *P. aeruginosa and K. pneumoniae* to PTZ in 1994–2019.

*Pseudomonas aeruginosa* (*P. aeruginosa*) and *Klebsiella pneumoniae* (*K. pneumoniae*) are among the leading pathogens responsible for deaths associated with resistance (Antimicrobial Resistance Collaborators, 2022). According to clinical trials and studies on pathogenic microorganisms, the antimicrobial sensitivity of PIP/TAZ against *P. aeruginosa* and *K. pneumoniae* gradually decreases over time (Figure 1) (Stobberingh et al., 1994; Fass et al., 1996; Kuck et al., 1996; Pelak et al., 2002; Pascual et al., 2007; Eagye et al., 2009; Hui et al., 2011; Sader et al., 2018a; Sader et al., 2018b; López-Jácome et al., 2022).

When using low doses, due to faster renal clearance in children, the blood drug concentration of PIP/TAZ rapidly decreases, making it often difficult to achieve the expected therapeutic effect (Kearns et al., 2003; Filler et al., 2021; Iacobelli and Guignard, 2021). However, high doses without pharmacokinetic evidence are also not advisable, which may be related to potential kidney injury in children (Tang Girdwood et al., 2023). After all, piperacillin ranks second in the FDA Adverse Event Reporting System (FAERS) as a drug associated with pediatric kidney injury (Zhang et al., 2023).

Considering the rapid development of renal clearance in children, the increase in bacterial resistance to PIP/TAZ, potential renal injury at high doses, and the differences in concomitant medication for different diseases, the potential treatment effect of PIP/TAZ cannot be assessed accurately based on empirical dosing. At present, there is still limited research on whether the PIP concentration in children with lower respiratory tract bacterial infection under the clinical medication regimen can meet the treatment needs. Therefore, our study aims to evaluate whether the current PIP/TAZ dosing regimen used in clinical practice can provide effective treatment for lower respiratory tract bacterial infections in children.

## 2 Patients and methods

### 2.1 Patients

A prospective study of PIP/TAZ was conducted at Shandong Provincial Qianfoshan Hospital and Hebei Children’s Hospital from 2015 to 2019. Patients older than 1 month and under 18 years of age, with suspected or proven lower respiratory tract bacterial infection, receiving PIP/TAZ as part of their regular antimicrobial therapy were enrolled in the study. The exclusion criteria were as follows: the estimated lifetime of patients could not support the whole treatment cycle; patients received systemic treatment with other investigational drugs; patients with other factors considered inappropriate by researchers for inclusion; for example, they require extracorporeal circulation or renal replacement therapy as co-existing medical conditions, have difficulties to collect blood samples, or were allergic to PIP/TAZ. This study conformed to the legal requirements and the Declaration of Helsinki and was approved by the Ethics Committees of Shandong Provincial Qianfoshan Hospital and Hebei Children’s Hospital, and written informed consent was obtained from the parents or guardians of the patients.

### 2.2 Sample collection and determination

Piperacillin sodium/tazobactam sodium (Qilu Pharmaceutical Co., Ltd.) was administered intravenously via injection for a duration of no less than 0.5 h, q6h–q12h daily. The dose of administration was expressed as the dose of PIP. One blood sample was collected from each patient after at least three doses of medication (at steady state). The blood sample collection time was ultimately evenly distributed throughout the entire dosing interval. Plasma was separated by centrifugation at 4,000 rpm for 10 min at room temperature immediately after sample collection and then stored at −80°C before analysis. PIP plasma concentrations were determined using the high-performance liquid chromatography method with ultraviolet detection (HPLC-UV) (Shimadzu LC - 2030C). The protein precipitation method is used to remove protein impurities from PIP plasma, with 210 μL acetonitrile, 5 μL 0.1 M hydrochloric acid, and 10 μL internal standard (sulbactam) added into 100 μL PIP plasma. The supernatant containing PIP was then extracted with 500 μL dichloromethane after removing protein impurities. Subsequently, 10 μL of the supernatant was placed in an autosampler at 4°C and injected into the HPLC system. The chromatographic separation was achieved by an Inert Sustain C18 column (5 mm, 4.6 * 250 mm; Shimadzu) at 35°C, with a mobile phase consisting of acetonitrile and 0.02 mol/L phosphate buffer with gradient elution at a flow rate of 0.8 mL/min. The UV wavelength was 218 nm. The detection range of PIP was 0.1–400 mg/L with a correlation coefficient value higher than 0.999. The method was proved to be accurate (% bias <7.3) and precise (CV < 9.1%) on an intra- as well as an inter-day basis. The lower limit of qualification (LLOQ) was 0.1 mg/L. For more intuitive comparison with reference lines, data below LLOQ were viewed as 0.1 mg/L.

### 2.3 PK/PD targets and MIC breakpoints

The individual predicted concentration of PIP was evaluated using the individual empirical Bayesian estimate method and NONMEM 7.4 software based on the previous population PK model of PIP (Nichols et al., 2015). The concentrations at the actual blood collection time point, 50% × *f*T, 70% × *f*T, and the time point when the minimum concentration (C_min_) is reached (100% × *f*T) were predicted. The prediction error (PE) was calculated with the observed concentration and predicted concentration at the same time points with Eq. 1, and the proportion of patients with PE% within ±20% and ±30% was calculated.

$$PE\%=\left|\frac{C_{P\text{re}dicted}}{C_{Observed}}\right|\times 100\%.$$ (1)

The evaluated PK/PD targets included (1) 70% *f*T > MIC, the free PIP concentration remained above MIC at least 70% of the dosing interval, and (2) 50% *f*T > 4× MIC, the free PIP concentration maintained above 4× MIC at least 50% of the dosing interval. The achievement of PK/PD targets was evaluated by the predicted concentration. If the predicted concentration of a patient at time points of 70% *f*T or 50% *f*T was higher than the MIC or 4× MIC, it was considered that the patient achieved the PK/PD target. Free PIP concentration was calculated as 70% of the total predicted concentration, as approximately 30% of PIP is bound to plasma proteins (Thibault et al, 2017; EMC, 2021).

The MIC breakpoints of PIP for *P. aeruginosa* (16.0 mg/L) and *K. pneumoniae* (8.0 mg/L) provided by the European Committee on Antimicrobial Susceptibility Test 2017 (EUCAST 2017) (Testing E-ECoAS, 2023) were adopted for PK/PD target estimation. The probability of target attainment (PTA) was assessed by the proportion of patients who reached the PK/PD targets. A PTA ≥90% was defined as optimal.

Patients were divided into the infants’ group (patients aged 1 month to 1 year) vs. the children group (patients older than 1 year), as well as the q8h group vs. the q12 h group. The independent sample t-test was used to compare the significant differences in PIP dose and the C_min_ between the infants’ and children groups post-administration, as well as between the q8h and q12 h groups. Descriptive analysis and independent sample t-test were conducted using SPSS 26 software.

## 3 Results

### 3.1 Patients

A total of 57 patients were enrolled and completed the study. The demographic characteristics are presented in Table 1. The study population consisted of 38 male and 19 female patients with a median age of 2.26 years (range: 0.17–12.58 years). The median body weight was 13.00 kg (range: 5.50–60.00 kg). The median dosage of PIP/TAZ was 50.00 mg/kg (range: 28.07–75.00 mg/kg), daily q8h or q12 h. No patient administrated in q6h was enrolled. The median infusion duration was 0.83 h (range: 0.50–1.92 h). The median (first to third quartile, interquartile range [IQR]) concentration of PIP was 8.12 mg/L (IQR 0.43–46.27) with a range of 0.10–130.73 mg/L. The median serum creatinine was 28 μmol/L (range: 16–59 μmol/L). All patients were evaluated for normal liver function.

**TABLE 1:** Demographic characteristics (N = 57).

	Median (range)		
Characteristics	All	Infants	Children
Sex [N (%)]			
Male	38 (66.7)	7 (50.0)	31 (72.1)
Female	19 (33.3)	7 (50.0)	12 (27.9)
Age (years)	2.26 (0.17–12.58)	0.61 (0.17–0.99)	2.93 (1.13–12.58)
Weight (kg)	13.00 (5.50–60.00)	7.85 (5.50–10.50)	15.00 (8.50–60.00)
Dose (mg/kg)	50.00 (28.07–75.00)	50.00 (46.62–73.12)	62.50 (28.07–75.00)
Daily dose (mg/kg/day)	142.86 (75.00–224.29)	147.96 (108.11–163.64)	138.89 (75.00–224.29)
Serum creatinine (μmol/L)	28 (16–59)	20 (16–29)	31 (19–59)

Data are median (range), unless otherwise indicated.

### 3.2 PK/PD targets

**FIGURE 2:**
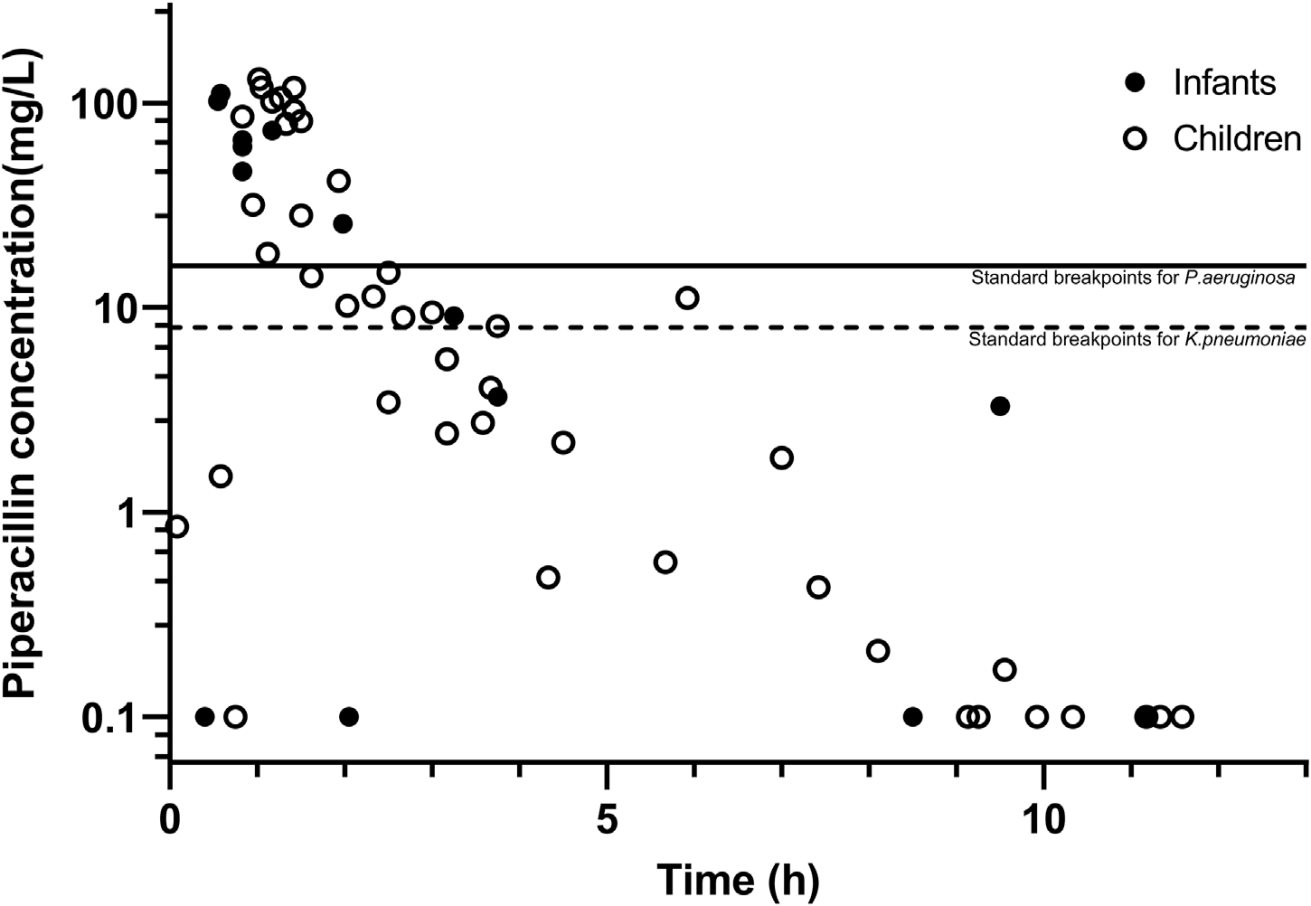
Plasma concentration–time curves of piperacillin, with the two lines representing the MIC of piperacillin against *P*. *aeruginosa* and *K*. *pneumoniae*.

**FIGURE 3:**
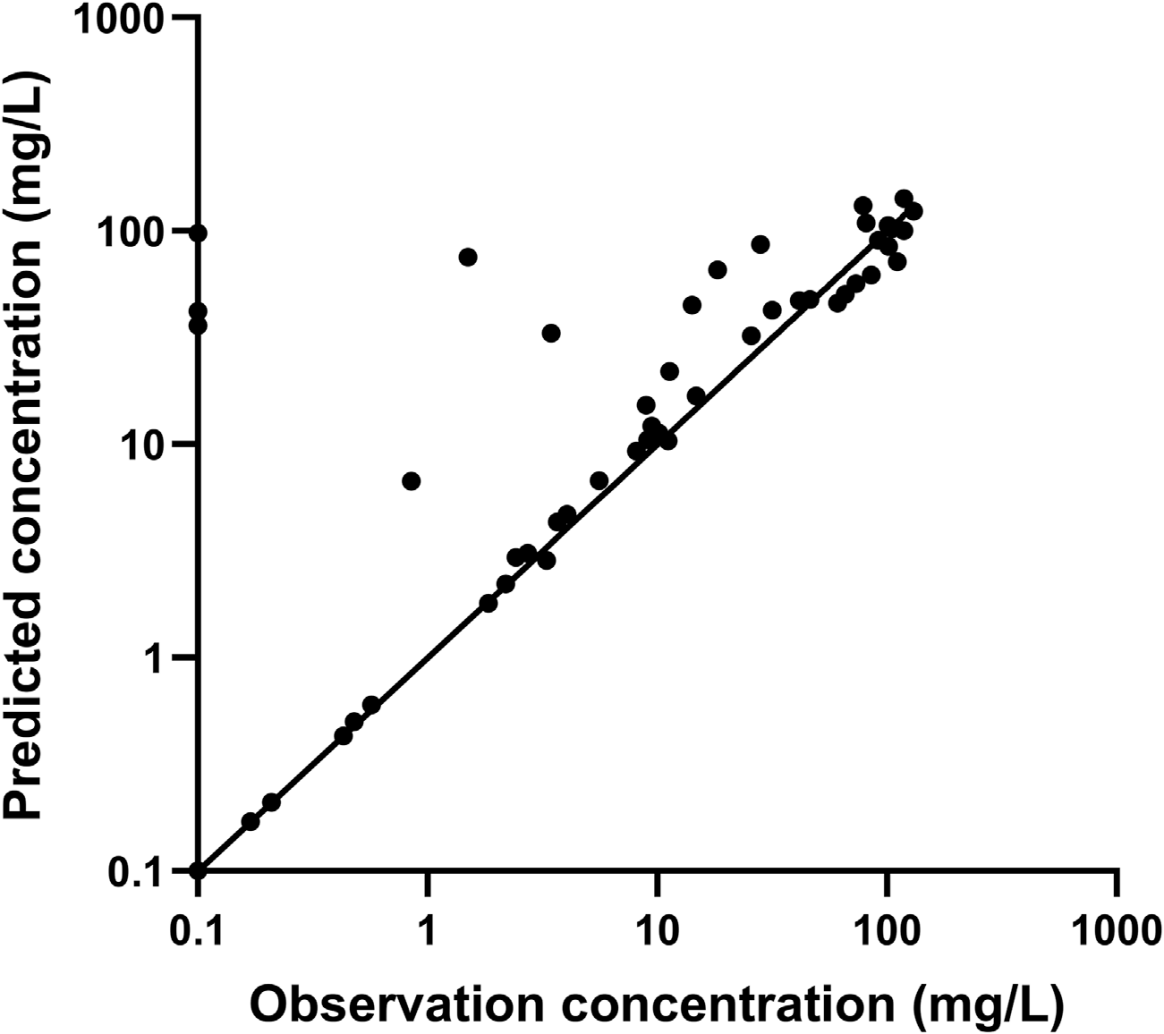
Fitting diagram of PIP observation and predicted concentration.

The PIP concentrations *versus* time are shown in Figure 2. The population PK model used for Bayesian estimation suggests that CL of PIP was only related to the body weight of children. The median PE% was 15.0%. The proportion of patients with PE% within ±20% and ±30% were 59.6% and 73.9%, respectively. The fitting diagram of PIP observation and predicted concentration is shown in Figure 3. The Bayesian estimation results indicate that no patients can meet the PK/PD targets, whether it was 70% *f*T > MIC or 50% *f*T > 4× MIC. The PTAs for the MIC of *P. aeruginosa* and *K. pneumoniae* were all 0.

We also compared the daily dose and C_min_ according to age and administration frequency in Table 2. There was no significant difference in PIP daily dose between infants (n = 14) and children (n = 43), but the median C_min_ of PIP was significantly higher in infants (0.36 mg/L) than in children (0.06 mg/L). Similarly, the median C_min_ value (0.43 mg/L) after administration in q8h (n = 24) was significantly higher than that (0.06 mg/L) after administration in q12 h (n = 33). The difference between the two groups of C_min_ was significantly higher than the difference between daily doses.

**TABLE 2:** Comparison of plasma concentrations between groups.

Groups		Number (%)	Daily dose (mg/kg/day)	*p*-value	Predicted C_min_ (mg/L)	*p*-value
Age	Infants	14 (24.6)	147.96 (108.11–163.64)	0.181	0.36 (0.06–4.68)	0.019
	Children	43 (75.4)	138.89 (75.00–224.29)		0.06 (0.00–2.51)	
Administration frequency	q8h	24 (42.1)	148.24 (84.21–224.29)	0.001	0.43 (0.00–4.68)	0.002
	q12 h	33 (57.9)	133.33 (75.00–150.0)		0.06 (0.00–0.50)	

Data are expressed as median (range), unless otherwise indicated. q8h, administered every 8 h; q12h, administered every 12 h. *p*-value, calculated by the independent-sample t-test. C_min_, the predicted minimum concentration after administration using the individual empirical Bayesian estimate method.

## 4 Discussion

The primary aim for giving antibiotic therapy is to achieve an efficacious and safe drug concentration and prevent the emergence of drug resistance in patients (Andersson and Hughes, 2014). For the last 25 years, the situation of antimicrobial resistance (AMR) of PIP/TAZ has worsened. It is becoming increasingly common that infections caused by antimicrobial-resistant bacteria are much more difficult to treat, sometimes even impossible to cure, and are associated with high hospitalization costs (Solomon and Oliver, 2014; Prestinaci et al., 2015; Arzanlou et al., 2017; Septimus, 2018). It is well known that AMR is associated with antibiotic misuse or overexposure to antibiotics, and the concentration of subtreatment antibiotics is often ignored (Rizk et al., 2017). There have been clear evidences to suggest that subtherapeutic concentrations can induce the development of AMR (Gullberg et al., 2011; Andersson and Hughes, 2014). When bacteria are exposed to subtherapeutic antibiotic concentrations, they are not immediately killed but can survive by increasing mutagenesis and/or recombination, thereby accelerating the evolution of drug resistance.

Under the clinical dose regimen, no patients can achieve the antimicrobial goals, indicating that the current dose regimen was far from meeting the therapeutic needs. According to package inserts of PIP/TAZ (Qilu Pharmaceutical, Jinan), clinical guidelines (Subspecialty Group of Respiratory Diseases, 2013), and reference books (Li-Juan et al., 2017; BNF Joint Formulary Committee, 2019), the recommended dosages of PIP/TAZ for pediatric patients range from 20 mg/kg to 120 mg/kg, twice to four times a day. The PIP/TAZ dose involved in this study may be determined by pediatric clinicians referring to this recommended dose, but it was still a long way from reaching the upper limit of this recommended dose. Regrettably, prescription of antibiotics at doses lower than recommended doses was very common in pediatric patients (Wang et al., 2021; Akkawi et al., 2022), which may be related to the conservative medication principles of pediatric clinicians. Due to the regulatory requirements of the Chinese drug regulatory authorities on the clinical use of antibiotics and the vulnerability of pediatric patients, pediatric clinicians often adopt low-dose and long-interval antibiotic administration plans when it is not possible to obtain timely medication evidence such as blood drug concentration and antibiotic resistance patterns. This strategy may fully consider the safety of pediatric patients but often overlooks the risk of AMR caused by subtherapeutic concentrations at low doses.

At the same dosage, the C_min_ in infants was significantly higher than that in children. PIP is mainly excreted through the kidneys (Li et al., 2020), and the renal function in infants is significantly weaker than that in children (Kearns et al., 2003; Filler et al., 2021; Iacobelli and Guignard, 2021). Considering that the blood drug concentration of PIP cannot meet the PK/PD targets in both infants and children, it is recommended to increase the clinical dosage of PIP/TAZ, especially in children. When the frequency of administration was q8h, the C_min_ of PIP was significantly higher than q12 h. Although neither of these administration methods achieved ideal antibacterial effects, it also suggests that shortening the dosing interval, extending infusion time, or continuous infusion may achieve better antibacterial effects (Thibault et al., 2017; Maarbjerg et al., 2019).

There are still some limitations to this study. First, although we have validated the inadequacy of current clinical treatment doses for PIP in children, a larger sample size is still needed to verify the universality of this issue. In future studies, more research centers and more patients will be included to investigate the universality of subtherapeutic concentration and the AMR caused by subtherapeutic doses, as well as the improvement of subtherapeutic concentration after pharmaceutical interventions. Second, this study discussed the current clinical treatment options from the perspective of pharmacokinetics. However, a more rigorous evaluation of the effectiveness of the current PIP/TAZ dosing regimen and the increase in AMR is needed in the control group receiving alternative treatment or placebo. This is also where further research needs to be improved. Additionally, due to the particularity of pediatric patients, we were unable to conduct long-term follow-up and did not collect any adverse reaction information from the patients. This may to some extent affect the evaluation of the therapeutic response and risk of lower respiratory tract infections with PIP subtherapeutic concentrations. Considering the current situation where continuous infusion is difficult to maintain in infants and children, more optimized drug delivery strategies or better alternative treatments need to be further explored. However, more importantly, it is necessary to consider how to provide evidence-based drug treatment strategies for pediatric clinicians to enhance their confidence in drug use. This is also a limitation of this study, but it is also a prospect for future research.

## 5 Conclusion

The current dose regimen of PIP/TAZ leads to extremely low plasma concentrations in most children with lower respiratory tract bacterial infections. The optimal dose regimen of PIP/TAZ needs to be investigated in the pediatric population using powerful developmental pharmacokinetic–pharmacodynamic study designs. It is necessary for clinical doctors to detect patterns of antibiotic resistance, which can enable treatment strategies to be adjusted accordingly to ensure optimal patient outcomes and minimize further development of resistance.

## Data Availability

The raw data supporting the conclusion of this article will be made available by the authors, without undue reservation.
